# Old and New Biomarkers of Alcohol Abuse: Narrative Review

**DOI:** 10.3390/jcm12062124

**Published:** 2023-03-08

**Authors:** Sara Fakhari, Napoleon Waszkiewicz

**Affiliations:** 1 Department of Psychiatry, Mazowieckie Specjalistyczne Centrum Zdrowia w Pruszkowie, 05-800 Pruszków, Poland; 2 Department of Psychiatry, Medical University of Bialystok, 15-089 Bialystok, Poland

**Keywords:** alcohol abuse, traditional biomarkers, new biomarkers, alcohol use

## Abstract

The harmful use of alcohol is responsible for 5.1% of the global burden of disease, and the early detection of alcohol problems may prevent its development and progression. Therefore, the aim of the study is to review traditional and new biomarkers associated with alcohol use. The nature and practical application and limitations of alcohol biomarkers in the diagnosis and monitoring of drinking are reviewed. Despite the limited specificity and sensitivity in alcohol drinking detection, traditional biomarkers are useful in clinical practice, and new generations of biomarkers, e.g., proteomic markers, are in need of further investigation. Traditional biomarkers are broadly available and cost-efficient, providing valuable data on the complications of drinking and prognosis, as well as on concurrent conditions affected by drinking. The most important challenge in the future will be to translate methodically advanced methods of detecting alcohol markers into simpler and cheaper methods. Larger population studies are also needed to test the usefulness of these potential markers of alcohol use.

## 1. Introduction

The harmful use of alcohol is responsible for 5.1% of the global burden of disease [1]. Approximately 2% to 4% of the population is diagnosed with alcohol dependence (AD). Around 20 to 30% of national healthcare costs and the same percentage of admissions to hospitals are due to alcohol use—it is found in 2/3 of patients in trauma wards [2,3]. Primary healthcare doctors manage to identify only 20% to 50% of alcohol addicts among patients who come to them for advice [4]. Among patients with mental health problems, over 20% of them abuse alcohol at some point in their lives [5].

From January 2022 onward, the followed classification in Europe has been ICD-11. In terms of alcohol dependence, there have been significant differences in those classifications.

### 1.1. ICD-10 (International Statistical Classification of Disease and Health Related Problems—Tenth Revision) 1996

The ICD-10 divides problems with alcohol overuse into acute alcohol poisoning, harmful use, alcohol dependence, etc. Suitable intervention demands the early and proper classification of a patient into the correct group of alcohol use (exception–abstinence). Such groups include:

- Social drinkers: Their drinking is not usually associated with health risks, and those who fall into this group have even reported a reduced risk of ischemic heart disease. People in this group consume no more than 1–2 (women) or 2–3 (men) standard units of alcohol per day.
- Risky drinkers: With these, there is a potential risk of health damage; they consume more than 1–2 (women) or 3–4 (men) standard drinks per day.
- Binge drinkers and heavy drinkers: The drinking of alcohol in those groups is on the verge of risky and harmful drinking.
- Alcohol-dependent persons: They feel compelled to drink, lose control of their drinking, have withdrawal symptoms, and possess high alcohol tolerance (reduced in the late phase). Drinking alcohol dominates over other behaviors that once had greater value. They continue drinking despite any evidence of harm (somatic, behavioral, social, and cognitive) [4,6,7].

### 1.2. DSM-5

The DSM-5 was established in 2013. The diagnostic categories of “alcohol abuse” and “alcohol dependence” were abandoned, and a new category of alcohol use disorder was created with a breakdown of the severity of the disorder: mild, moderate, and severe. The following degrees of severity of the disorder were distinguished:

- Mild (2–3 symptoms present)
- Moderate (4–5 symptoms)
- Severe (6 or more symptoms)

This is believed to be a step back from the dichotomous, zero–one understanding of alcohol dependence. It introduces an element of the dynamics of the disorder, the intensity of which may vary over time, ranging from spontaneous remissions to exacerbations. This is a response to the latest recommendations for alcohol dependence therapy.

### 1.3. ICD-11

The ICD-11 was put into service in January 2022.

**Figure 1:**
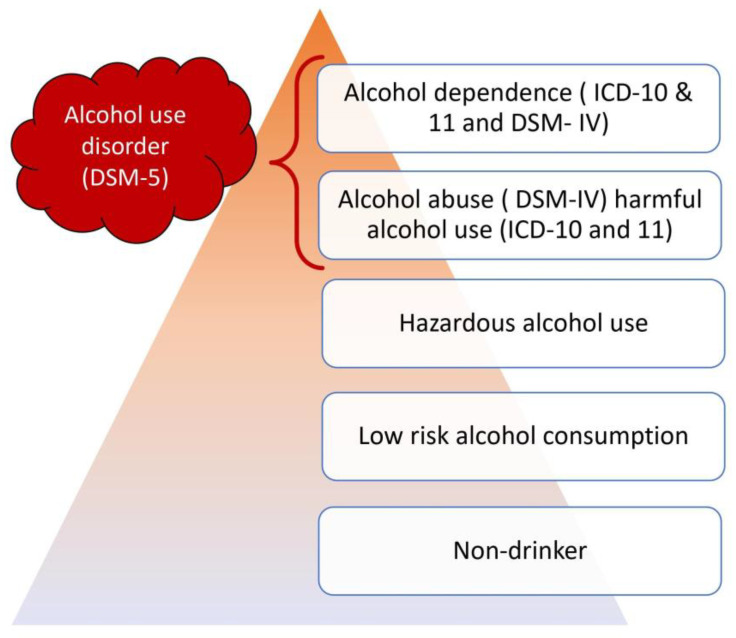
Differences and similarities in different classifications.

The division into harmful drinking and alcohol addiction has been maintained. Harmful drinking was extended to the occurrence of families harmed by substance use (Figure 1).

ICD-11 classification:

1. Single-episode use of alcohol
2. Harmful pattern of use of alcohol
  - Episodic (current use, episodic in the last year of symptoms, with intermittent heavy drinking (less than a month) but periods of abstinence from alcohol)
  - Continuous (alcohol dependence, current use, continuous drinking daily or almost daily for the last month or more)

3. Alcohol dependence (AD)

### 1.4. AD-Remission

- Full early remission (alcohol dependence, early full remission)—abstinence sustained from 1 to 12 months
- Partial remission—a period of over 12 months and a visible significant reduction in the amount of alcohol consumed, and although there are still periods of drinking alcohol, the general AD criteria are no longer met
- Full remission—abstinence for 12 months or more in a person with previously diagnosed AD

### 1.5. Other Disorders Related to Alcohol in ICD-11

4. Alcohol intoxication
5. Alcohol abstinence syndrome (uncomplicated, with perceptual disturbances, with seizures, with sensory disorders and seizures)
6. Alcoholic delirium
7. Psychotic disorders related to alcohol use (with hallucinations, with delusions, with mixed psychotic symptoms)
8. Other (alcohol-induced mood disorders, alcohol-induced anxiety disorders, amnestic syndrome, dementia syndrome)

### 1.6. Usefulness of Biomarkers

Complementing the diagnostic process and confirming abuse is the use of laboratory tests. The role of laboratory tests in alcohol diagnostics is as follows:

- They provide objective information about alcohol consumption and changes in consumption time
- Confirm the results obtained from the interview and questionnaire research
- They can be used as screening tools for doctors’ patients’ first contact in admission rooms and psychiatric, gynecological, and internal medicine wards
- They are very useful in situations where when it is not possible to collect anamnesis (unconscious patients, after physical injuries, post-mortem examinations),
- Useful for people who often hide their abuse (participating in traffic accidents, pregnant women)
- Useful in assessing the role of alcohol in the disease process;
- In the diagnosis and differentiation of disorders;
- In controlling the effectiveness of treatment and therapy;
- In the early recognition of drinking relapses;
- In forensic medicine (assessment of a person’s sobriety at the time of death);
- In prenatal diagnosis (assessment of fetal exposure to alcohol);
- In setting limits for safe alcohol consumption
- Provide information about the harmful effects of alcohol consumption and play a motivational role in changing the way of drinking to a less harmful one

## 2. Aims and Methods

As the harmful use of alcohol is responsible for more than 5% of the global burden of disease, and the early detection of alcohol problems may prevent its development and progression, the aim of the study is to review traditional and new biomarkers associated with alcohol use.

A literature search was conducted in the PubMed, Scopus, and Web of Science databases using the keywords ‘marker’, ‘alcohol’, ‘ethanol’, ‘alcohol marker’, ‘alcohol biomarker’, ‘ethanol marker’, and ‘ethanol biomarker’, as well as combinations of these terms. We included clinical studies, meta-analyses, reviews, and case studies regarding alcohol use detection, as well as potential alcohol markers.

## 3. Results (Markers)

### 3.1. Traditional Biomarkers of Alcohol Abuse

The US National Institute of Health defines a biomarker as “a characteristic that is objectively measured and evaluated as an indicator of normal biological processes, pathogenic processes, or pharmacologic responses to a therapeutic intervention” [8]. In the context of alcohol abuse, a biomarker is a precise indicator of one’s drinking pattern or any genetic predisposition toward alcohol abuse and addiction. Those two qualities of biomarkers are set off as state markers (i.e., evaluation of a patient’s history of alcohol consumption by biochemical measures) and trait markers (i.e., revealing one’s inherited risk of developing alcohol dependence due to chronic usage by biochemical tools) [9]. More importantly, diagnostic power is a way to identify the potential utility of a biomarker. Sensitivity (the diagnostic method has to be applicable to almost all users) and specificity (the diagnostic method has to be linked only to alcohol use but not any other problems/underlying conditions) are important aspects to consider when qualifying a biomarker as useful. Accuracy and precision are the most important from a practical perspective [10,11]. According to pathophysiology, state markers of excessive alcohol intake can be grouped into two types:

- Indicators of alcohol consumption (acute as well as chronic)
- Indicators of alcohol-induced organ damage.

Traditionally, with alcohol dependence, we link aspartate aminotransferase (AST), carbohydrate-deficient transferrin (CDT), mean cell volume (MCV), alanine aminotransferase (ALT), and γ-glutamyl transferase (GGT) [11,12,13]. Low specificity and sensitivity among different populations hamper their utility. However, it is worth emphasizing that over an extended period of time, the US FDA only approved CTD as a test [8,14,15].

### 3.2. Ethyl Alcohol

Measurements of the concentration of ethyl alcohol in the blood and in the exhaled air form the basis of intoxication diagnostics in emergency rooms. It is assumed that a routine blood alcohol concentration ≥ 1‰ (per mille), >1.5‰ without signs of intoxication and >3‰ in all circumstances, suggests an increase in tolerance and thus the possibility of alcohol addiction [2,16,17]. In relation to the rapid elimination of alcohol from the blood (approximately 1 g/1 h/10 kg), this test is useful in the short term (up to 6–8 h), especially for heavy drinkers who eliminate it about 1.5 times faster than nondrinkers or who go to a doctor after 24 h of abstinence [18,19,20]. Because of retention in the bladder, ethanol can even be detectable in the urine for several hours longer than in the blood [21,22,23]. It can also be detectable in saliva and sweat.

### 3.3. Gamma-Glutamyl-Transferase

Gamma-glutamyl-transferase (GGT) is an enzyme in many cell membranes involved in the transfer of γ-glutamyl residues to acceptors [2]. Its highest concentration is found in the cell membranes of hepatocytes and the epithelial cells of the bile ducts, from where it is released in case of damage [23,24]. GGT serum activity increases in 75% of alcohol addicts, in whom the daily dose of chronic alcohol consumption exceeds 40 g of pure alcohol per day. In individuals with alcoholic liver disease, GGT activity rises to even more than ten times the normal upper limit [25]. In non-addicted people and those who have not previously abused alcohol, GGT activity increases only after a 5-week period of consuming >60 g of alcohol/day [4]. GGT activity is age-dependent, hence its little growth and its usefulness in people under 30 years of age. The limited usefulness of GGT in the diagnosis of alcohol problems has been shown among women and members of the binge drinkers group [26,27,28,29]. The half-life (T½) of GGT ranges from 14 to 26 days, returning to normal after about 4–5 weeks [4]. GGT can produce false positive results in cases of biliary tract diseases, non-alcoholic liver diseases, obesity, diabetes, hypertension, pancreatitis, hyperlipidemia, hyperactivity of the thyroid gland, after serious injuries, in inflammatory processes, blood clots and embolisms, heart and kidney diseases, in the course of treatment with barbiturates, benzodiazepines (BDZ), tricyclic antidepressants (TCAs), anticonvulsants (carbamazepine, phenytoin), anticoagulants, non-steroidal anti-inflammatory drugs (NSAIDs), and in people who smoke cigarettes [2,4]. Despite not having high specificity (50–72%), GGT is now the most commonly used marker of alcohol abuse, especially in confirming a clinical diagnosis of AD and monitoring abstinence during treatment [2].

### 3.4. Aspartate and Alanine Aminotransterase

An increase in the activity of aspartate aminotransferase (AST) and alanine aminotransferase (ALT) in serum to about 2–4 times above normal is common among people addicted to alcohol [4]. The sensitivity of the tests varies from 25–60% for AST and 15–40% for ALT. Non-recurring drinking of a small amount of alcohol usually does not increase the activity of transaminases; however, a higher dose of 3–4 g/kg may increase AST values within 1–2 days, even in healthy people [4,24,30]. In patients with alcoholic hepatitis, a 5–10-fold increase in AST activity above the normal upper limit is usually seen [25]. As screening tests, aminotransferases are less sensitive than GGT in detecting heavy drinking [24]. The increase in the value of transaminases depends more on the degree of liver damage rather than alcohol consumption per se; however, the enzyme increase depends on the level of alcohol consumption (including the recent one) [2,4]. Plasma AST values do not correlate with the time of alcohol drinking, although higher levels of AST are observed in addicts with a history of drinking and dependence of more than 10 years [27]. ALT is a cytosolic enzyme that is more specific than AST in liver disturbances [25]. A total of 80% of AST activity is located in the mitochondria (mAST), with 20% in the cytosol (cAST) of hepatocytes, and extrahepatic activity is high in the skeletal muscle, heart, kidneys, brain, and pancreas [24]. Aminotransferases catalyze the reaction of the transferring of amino groups from amino acids to α-ketoacids, which requires the action of the coenzyme–pyridoxal phosphate (a derivate of vitamin B6) [25]. Pyridoxal phosphate deficiency in the course of alcohol dependence inhibits ALT activity, hence causing an increase in AST activity. This phenomenon was used to determine the De Rittis Index (AST/ALT), in which a value >1.5 suggests damage, and >2 is almost a confirmation of the alcoholic etiology of a damaged liver, which is very useful for distinguishing alcoholic liver damage from non-alcoholic liver damage [2,4,31]. Additionally, a greater increase in serum AST may depend on severe damage to hepatic mitochondria in which most AST is found, as well as the half-life of enzymes (in blood, T½ for ALT is 47 h, ~17 h for total AST activity, and about 87 h for mitochondrial AST) [25].

### 3.5. Mean Corpuscular Volume

An increase in the mean volume of red blood cells (MCV–Mean Corpuscular Volume) occurs in 4% of the adult population, 65% of which is related to alcohol abuse [2]. It is estimated that macrocytic anemia originates from the direct hematotoxic effect of ethanol and its metabolites, which also increases the permeability of erythrocyte cell membranes (changes in the protein-lipid structure), disrupts the structure and metabolism of cells (the formation of acetaldehyde adducts with cell proteins and membranes), and increases the susceptibility of erythrocytes to damage and hemolysis (T1⁄2 shortening) [2]. Folic acid deficiency is also expected to be the cause of an MCV increase [4,23,24]. The possible false positive increases in MCV include vitamin B12 and folic acid deficiency, hypothyroidism, hemolytic disease with reticulocytosis, non-alcoholic liver disease, rises with age, use of anticonvulsants (e.g., phenytoin), zidovudine, azathioprine, and smoking [4]. Heavy drinkers commonly show an increase in MCV after drinking more than 60 g of alcohol a day for a minimum of one month; this rise corresponds with the amount and frequency of alcohol consumption [4]. Nevertheless, constant drinking, even in small amounts (<40 g/day) may increase MCV values by 1–2 units when compared to abstinence [2]. Owing to the long lifetime of erythrocytes (healthy–about 120 days, damaged–slightly shorter), it takes about 2 to 4 months of alcohol abstinence to return to normal MCV values [2]. Hence, MCV is not used to monitor abstinence and the relapse of drinking [4]. The sensitivity of MCV ranges from 40–50%, with a relatively high specificity of 80–90% (higher than the specificity of GGT) [4,24]. The utility of MCV was shown in alcohol abuse by women (higher sensitivity of the test than in men), especially in the group of heavy drinkers, and also in the screening of the risk of alcohol damage to the fetus (FAE–Fetal Alcohol Effects) [2,4,24].

### 3.6. Carbohydrate Deficient Transferin

Low-carbohydrate isoforms of transferrin (CDT–Carbohydrate Deficient Transferrin), i.e., desialized transferrin, is a well-known, highly sensitive, and specific test (82% and 97%, respectively) in the diagnosis of alcohol dependence [4,32]. Whilst desialylated transferrin is a fairly new marker of alcohol abuse, its usefulness in diagnostics is increasing [2,26,29]. As an only test, it has been approved in the USA by the FDA (Food and Drug Administration) in identifying heavy drinking [20]. Ethanol and its metabolites may lead to the dysfunction of enzymes liable for the modification of transferrin (increase in sialidase activity, decrease in sialyltransferase) and disrupt the functions of hepatic cell receptors responsible for the elimination of desialated transferrin [2]. CDT includes transferrin with a reduced amount of sialic acid (asialo-, monosialo-, and disialotransferrin) [33]. Drinking between 50 and 80 g of alcohol daily for a minimum of 1 week or >60 g/day for 7–10 days significantly increases the level of CDT in the serum, and after a short period of abstinence, even small amounts of alcohol can significantly increase the serum level again [4,32]; therefore, CDT is considered a more sensitive test than GGT in detecting relapse drinking (abstinence monitoring) and differentiating alcoholic from non-alcoholic liver injuries. CDT shows low sensitivity (12–45%) in the general population, women, young people, binge drinkers, and healthy people, even at high doses of alcohol [4]. As a screening test in the general population, CDT is not more sensitive than GGT, so its sensitivity relates to the level of CDT and the total level of transferrin (% CDT; CDT ratio/total transferrin) [4]. CDT levels return to normal within few weeks of abstinence, with T1⁄2 ~ 15 days [4]. Certain diseases reduce the specificity of the CDT test. These include non-alcoholic liver diseases (primary biliary cirrhosis, chronic active inflammation, -HCV, hepatocellular carcinoma), senile dementia, depression, pregnancy, solvent poisoning, genetic glycoprotein deficiency syndrome, pancreatic and kidney transplantation, cystic fibrosis, insulin-related metabolic disorders, iron deficiency, galactosemia, and anal cancer [4,34]. A study based on saliva found that salivary CDT, unfortunately, is rather inapplicable as an alcohol marker [35] (Table 1 and Table 2).

### 3.7. Combinations of Biomarkers

When monitoring abstinence among patients diagnosed with alcohol problems, CDT and GGT tests are the most useful. The combination of two or three markers seems to be the most optimal, especially the use of GGT with CDT (the so-called γ-CDT), which significantly increases the sensitivity of identifying alcohol abusing patients in various clinical conditions without reducing the specificity of the tests [2,24]. This combination makes it possible to determine not only the amount of alcohol consumed and the severity of liver damage (CDT or GGT) but also the frequency (CDT) and intensity of drinking (GGT) [26]. Another combination may be CDT + MCV, although it may be less readily accepted [4] (Table 3).

### 3.8. New Biomarkers of Alcohol Abuse

#### 3.8.1. Ethyl Glucuronide (EtG) and Ethyl Sulfate (EtS)

Ethanol created in reduced quantities, mainly in the endoplasmic reticulum of the liver, has straight conjugated metabolites: ethyl glucuronide (EtG) and ethyl sulfate (EtS). Compared to ethanol examination, EtS and EtG are premium indicators of current alcohol intake with a longer recognition span. In particular, EtG exists in the blood for approximately 36 h (in comparison with the complete elimination of ethanol from the blood after 8 h) and exists in urine for 3–5 days after hefty alcohol intake, whereas EtS is noticeable in urine c.a. 16–27 h longer than ethanol [39]. When individuals test positive for EtG, it is most likely that they were drinking alcohol, even if there is no ethanol detected. This is why EtG is specifically helpful for identifying alcohol consumption, specifically in alcohol addiction therapy programs. Along with urine and blood, EtG can also be spotted in various other body liquids, body tissues, and hair [9,13,48]. A couple of studies have actually shown that EtG measurements in hair have quite a high sensitivity and specificity in the recognition of alcohol abuse: 80–95% and 70–90%, respectively. Notably, EtG and EtS outcomes need to be translated in the context of all readily available medical and behavioral info. It has actually been reported that incidental exposition to alcohol (e.g., hand sanitizers, mouth wash) might lead to EtG and/or EtS detection. On top of that, upper respiratory system infections along with β-glucuronidase hydrolysis may reduce EtG levels; however, they do not appear to impact EtS. Additionally, extra EtG in hair is prone to aesthetic therapies [49]. An additional weak point of EtG as a biomarker of alcohol abuse is the rather innovative approach needed for a precise analysis of EtG in urine. Hence, many attempts to create a step for easier urine-based EtG methods or to determine EtG in various other body liquids or hair have actually produced less-than-acceptable outcomes [9,13,50].

#### 3.8.2. Acetaldehyde, Acetaldehyde Adducts, and Anti-Adduct Antibodies

Ethanol’s oxidative metabolism, as an initial result, produces acetylaldehyde. By binding with numerous proteins, including albumin, hemoglobin, and many other RBC membrane proteins, serum proteins, CYP450 2E1, it causes acetaldehyde-protein adduct formation. Complying with alcohol consumption, a concentration of free acetaldehyde is very changeable with a life expectancy of c.a. 3 h; however, some acetaldehyde-protein adducts might be found approx. 3 weeks after alcohol intake, and hemoglobin-bound acetaldehyde (HAA) gathers in red blood cells during their 120-day typical lifespan. A singular high amount of alcohol (2 g/kg) boosts blood HAA when traditional markers, such as GGT or MCV, reveal no difference [41,51]. Methods focused on identifying both free as well as bound acetaldehyde in blood are actually established. A designated entire blood-associated acetaldehyde assay (WBAA) has the potential to be an incredibly sensitive and specific tool to evaluate for alcohol intake and relapses in alcohol addiction treatment programs. The capability of HAA or WBAA assays to determine alcohol usage ensamples in time make them distinct amongst alcohol biomarkers.

Biomarkers of alcohol consumption can be used in circulating antibodies against acetaldehyde adducts. When antibodies bind with protein, acetaldehyde produces a molecular adduct. This adduct contains acetaldehyde in the form of hapten, which is why it develops a neo-antigen that can generate autoantibodies [13,51]. In heavy drinkers and alcohol-dependent people (but not in social drinkers), a raised sensitivity of IgA with acetaldehyde-modified proteins has been reported. Moreover, a raised proportion of IgA/IgG is extremely suggestive of alcoholic liver disease [13]. Nevertheless, the levels of specificity and sensitivity of the circulating anti-adduct antibodies are 65–73% and 88–94%, respectively [41]. Surprisingly, a singular high dose of alcohol (2 g/kg) has indeed been indicated to increase the amount of salivary IgA. As saliva is a conveniently and non-invasively acquired fluid, salivary anti-acetaldehyde adducts Ig As appear to reveal promise in binge drinking recognition [40].

#### 3.8.3. Fatty Acid Ethyl Esters

The non-oxidative metabolism of ethanol produces fatty acid ethyl esters (FAEEs). These compounds are formed due to the conjugation between fatty acyl chains (such as palmitic acid, oleic acid, and stearic acid) and ethanol. Enzymes (such as FAEE synthase, microsomal acyl-CoA: ethanol o-acyltransferase, carboxylesterase, lipoprotein lipase, cholesterol esterase, and triglyceride lipase) catalyze this reaction, but FAEEs can also be formed impromptu [9]. These metabolites are shown throughout the body, e.g., in the liver, pancreas, brain, heart, blood, WBC, adipose tissue, meconium, and most readily in hair and fat [49,52]. Ethanol is distributed by the formation of FAEE and acetylaldehyde. Fat ethyl esters are used as a postmortem indicator of alcohol consumption. Animal and human studies indicate that FAEEs measured in fat can serve as a marker of alcohol consumption for up to 12 h after death, while those tested in liver cells may work for about 24 h after alcohol consumption [53]. This kind of application is desirable because traditional markers, e.g., blood alcohol levels, may be artificially elevated due to postmortem alcohol formation. Moreover, FAEEs found accumulated in the hair are a very promising marker of a person’s drinking habits because they cannot be rinsed out of the hair and accumulate with alcohol consumption. Interestingly, FAEEs have certainly been found to accumulate in the proximal 5–10 cm of hair and then decrease to a plateau regardless of the amount of alcohol consumed. Thus, through segmental hair assessment, it seems possible to confirm a current period of prolonged abstinence if FAEE levels corresponding to significant alcohol exposure are not observed in the freshly grown portions [53]. Separate compounds (stearate, ethyl myristate, palmitate, oleate) appear to be detailed and sensitive markers of chronic excessive alcohol consumption in adults, differentiating social drinkers from heavy drinkers or alcohol addicts [54,55]. At the appropriate cut-off level (0.5 ng cumulative FAEEs per mg of hair), it was found to have 90% sensitivity in detecting alcohol abuse. Hair FAEE levels between 0.2 and 0.5 ng/mg have been found to be indicative of social use, often overlooking abstinence, while levels above 1.0 ng/mg are almost 100% specific to heavy alcohol consumption; however, they are less sensitive (~ 75%) [55,56]. Note that cosmetic treatments and hair care can have an effect on FAEE in the hair, although preliminary research seems to suggest that these effects are of little scientific importance [57]. Additionally, FAEEs, through their measurement in meconium, have a clear value as a reliable test to provide evidence of prenatal alcohol exposure in neonates due to drinking during pregnancy [58,59].

#### 3.8.4. Phosphatidylethanol

Phosphatidylethanol (PEth) is an example of an unusual cell membrane phospholipid formed only in the presence of ethanol. Although the phospholipase D catalyzed reaction has been detected throughout the body, for the use of PEth as a biomarker of alcohol consumption, it was tested in blood cells, where it was most readily obtainable and measurable [60]. In vitro studies showed that the amount of PEth in human red blood cells was directly proportional to ethanol concentration and exposure time, and there was no correlation between the rate of PEth formation and hematological indices (i.e., MCV, red blood cell count, hematocrit). Moreover, there is no enzymatic degradation of PEth in human erythrocytes, so it is deposited in cell membranes, suggesting a potential utility in measuring long-term or binge alcohol consumption. Importantly, PEth is believed to be less sensitive than EtG or EtS to small amounts of ethanol and does not detect single episodes of drinking [60]. The limit of total ethanol consumption resulting in a positive PEth test was set to c.a. 1000 g in 3 weeks, with a daily consumption of at least 50 g [61]. As PEth formation is especially ethanol-dependent, the diagnostic uniqueness of PEth as an alcohol biomarker is theoretically 100%. Interestingly, its sensitivity was found to be high, between 94.5 and 100% [62,63]. Unlike traditional indirect biomarkers used to diagnose chronic drinking behavior (i.e., AST, MCV, ALT, CDT, and GGT), blood PEth does not appear to be influenced by age, gender, other substances consumed, or a lack of alcohol, comorbidities such as kidney disease, liver disease, and high blood pressure. Unlike EtG or EtS, PEth is considered insensitive to accidental exposure to ethanol, such as via mouthwash and antibacterial hand washes [61,62]. The usefulness of PEth in the medical diagnosis of alcohol abuse is determined by the short-term nature of this marker; its mean half-life in the blood of addicts is about 4 days (3–5.3 days). In clinical trials, this compound was detectable in the blood of chronic drinkers for up to 28 days after being sober [61]. Despite such high efficiency, the existing approaches to detecting PEth are still too difficult for standard scientific application, although they can be effectively measured in nanomolar units in blood.

#### 3.8.5. β-hexosaminidase

β-hexosaminidase (β-HEX) is a lysosomal exoglycosidase present in most cell types and will harvest participation in the catabolism of glycoproteins, proteoglycans, etc., by releasing N-acetylhexosamines from the non-reducing end of their glycoconjugate oligosaccharide chains [13,45,64]. The consumption of large amounts of alcohol, i.e., >60 g per day for at least 10 days in a row, causes marked changes in enzymatic activity in body fluids. One of the proposed mechanisms of this change is lysosomal damage, which then causes the leakage of the enzyme from lysosomes and cells into body fluids [45]. It was established that the diagnostic sensitivity levels of increased β-HEX B activity in serum and β-HEX in urine are 69–94% and 81–85%, respectively. In addition, in alcohol-dependent people, the level of enzymes drops dramatically during the sobriety period (7–10 days, T½ = 6.5 days) [64,65]. However, levels of β-HEX in saliva, urine, or serum may also increase after isolated ingestion of about 2 g/kg of alcohol (also known as ‘binge drinking’) [66]. Despite the relatively high specificity (84–98%), people with liver disease (such as cirrhosis and cholestasis), thyrotoxicosis, diabetes, hypertension, pregnancy, myocardial or brain infarction, and those who take oral contraceptives may exhibit a false positive result due to the elevation of enzyme activity [64,65]. The unique advantage of β-HEX as a probable marker for prolonged alcohol abuse is that it is an inexpensive and simple detection technique. Other lysosomal exoglycosidases have also been found to be possible alcohol markers, e.g., isoenzyme A, β-hexosaminidase (HEX A) as a marker of chronic alcohol consumption intensity, α-fucosidase (FUC) and α-mannosidase (MAN) as markers of alcohol dependence, and β-glucuronidase (GLU) as a marker of both these states simultaneously (intensity + relationship) [42].

#### 3.8.6. Plasma Sialic Acid Index of Apolipoprotein J

The term “Plasma Sialic Acid Index for Apolipoprotein J” (SIJ) is understood to mean the ratio of moles of sialic acid per mole of apolipoprotein J (Apo J). Apolipoprotein J (another word clusterin) is a multifunctional N-glycoprotein found in high-density lipoprotein (HDL) complexes that has been linked through a variety of pathological and physiological processes. It is believed to be involved in the transfer of lipids between lipoproteins, especially cholesterol. The glycoprotein is highly sialylated, i.e., human Apo J has been shown to consist of 28 moles of sialic acid residues per mole of Apo J, compared with 4–6 moles of sialic acid per mole of transferrin [13]. This is very important and can help identify changes in sialic acid composition caused by alcohol use. As with molecular transferrin, long-term ethanol poisoning reduces plasma Apo J sialylation, primarily by increasing sialidase activity and reducing cellular glycosyltransferases (i.e., [67]). SIJ is reduced in alcoholics (typically by 50–57%, with a specificity of ~100%), and SIJ levels gradually return to a normal range over the course of several weeks of abstinence from alcohol (T½ = 4–5 weeks) [63,68]. Moreover, plasma SIJ is associated with relapses in people addicted to alcohol (sensitivity ~ 90%) [13]. Despite the fact that full scientific studies are still lacking, and we need more of them to be 100% certain, preliminary findings show that SIJ is a certain marker of alcohol abuse. However, for it to be widely used, there is a need to simplify the method for the determination of sialic acid in Apo J plasma. Currently, it can only be performed in specialized laboratories, which reduces the ease and availability of testing.

#### 3.8.7. Total Serum Sialic Acid

Glycoproteins, e.g., glycolipids, Apo J, and transferrin, are predominantly attached to serum sialic acids in people. Aside from these two fractions of sialic acid, total serum sialic acid (TSA) will also contain a small fraction of serum free sialic acid (FSA) produced by glycoprotein desialylation. Serum TSA and FSA levels from excessive alcohol consumption appear to be affected by changes in most sialylated glycoproteins [69]. News and information from the specialist literature have indeed shown that TSA concentration has great potential as a marker of massive alcohol consumption. Compared to social drinkers, alcoholics have increased amounts of TSA in saliva, urine, and serum, although the exact mechanisms by which it occurs are debated [13,69,70]. The diagnostic value of TSA as a biomarker of alcohol abuse showed a level of sensitivity of 48–58% and a specificity of 64–96% [13,69]. Unfortunately, various conditions and diseases, such as pregnancy, diabetes, cancer, cardiovascular disease, and kidney disease increase the serum TSA concentration, reducing its specificity [71]. Nevertheless, in a research context, TSA levels did not differ in people with typical and elevated liver enzymes, unlike the lipid-associated portion of sialic acid [69]. Despite its lack of specificity, TSA can be considered a good test for alcohol abuse, regardless of the presence of liver cell damage. The TSA test may not be handy in therapeutic programs to evaluate patients for relapse, as TSA levels persist longer than GGT or CDT and decline with abstinence.

Interestingly, preliminary studies have shown elevated serum FSA levels in alcohol-dependent individuals. Diagnostic accuracy reached 85–94%, although the sensitivity level was low (~40%). Compared to conventional markers of alcohol abuse, the scientific utility of FSA is significantly lower than that of GGT and CDT, although the specificity and predictability of these tests were similar. Currently, the scientific relevance of FSA is limited to inherited diseases, including Salla’s disease, childhood sialic acid storage disease sialidosis, and neuraminidase deficiency [44,69].

#### 3.8.8. Cholesteryl Ester Transfer Protein

Hydrophobic glycoprotein, also known as the cholesteryl ester transfer protein (CETP), is synthesized in liver cells. It circulates in plasma and is mainly bound to HDL molecules. It causes the redistribution of cholesteryl esters, phospholipids, and triacylglycerols among lipoproteins [13]. According to the literature, CETP activity and its plasma concentration are minimized by alcohol consumption. This leads to a diversion of the cholesteryl esters and an increase in plasma HDL levels (a frequent laboratory peculiarity in people addicted to alcohol) [13,28]. The potential scientific value of CETP in plasma is believed to be similar to traditional alcohol markers such as AST, ALT, GGTP, and MCV. However, its specificity is limited due to some aspects affecting the plasma level (e.g., disease diversity, dietary differences, medications) [13].

#### 3.8.9. 5-Hydroxytryptophol, 5-Hydroxyindole-3-Acetic Acid

One of the secondary metabolites of the hormone and neurotransmitter serotonin, 5-Hydroxytryptophol (5-HTOL), is a common component of urine. Alcohol and the main metabolite of its oxidation, acetaldehyde, affect the metabolism of serotonin in such a way that the level of 5-HTOL rises after drinking alcohol. Elevated levels of 5-HTOL persist in urine for 5–15 h (depending on the dose) after alcohol consumption, compared to baseline measurements that suggest that ethanol persists longer in urine than in blood [37]. Preliminary studies suggest that urine 5-HTOL screening is both sensitive and specific in detecting recent heavy alcohol consumption and may prove particularly useful in forensic toxicology. In addition, it is possible to use the test to monitor the abstinence of people participating in abstinence/treatment programs (except for those treated with disulfiram, which can also increase 5-HTOL levels) [37].

It is believed that the ratio of 5-HTOL to another serotonin metabolite, 5-hydroxyindole-3-acetic acid (5-HIAA), is a variable marker that can be verified for ethanol content in the body [65]. The 5-HTOL: 5-HIAA ratio was found to have 100% sensitivity up to 4 h after a medium dose of ethanol, but its reliability decreased after 7 h [72]. The short duration and advanced assay methods limit the diagnostic usefulness of these markers for the assessment of past ethanol abuse and make it difficult to translate them into clinical practice [65].

#### 3.8.10. Salsolinol

Salsolinol is a chemical compound made in the brain and other tissues as a condensation product of dopamine with acetaldehyde after alcohol consumption (it may also be the result of an enzymatic reaction between alcohol and pyruvate (a glucose metabolite that is used by cells as an energy source) [9]). It can be used as a marker of alcohol abuse. The chemical structure indicates that it is a biologically active alkaloid. It has an effect similar to morphine [73]. The usefulness of salsolinol as a potential marker of persistent alcohol consumption depends largely on the tissue as well as the method of its determination [16]. It has been shown that the total amount of salsolinol in urine and the concentration of salsolinol in plasma change differently after acute alcohol consumption. Alcohol consumption showed a significant increase in both urine and plasma levels. Moreover, compared with non-alcoholics, alcoholics who abstained for only 1 week had decreased levels of salsolinol in one type of white blood cell (especially lymphocytes) [74]. On the other hand, studies on salsolinol levels in the brain have found no differences in salsolinol levels between alcoholics and non-alcoholics [16]. Several studies have shown that salsolinol can be formed from nutrients (e.g., bananas) and has a major influence on plasma levels [75].

Due to the low uniqueness and the possibility of residual alkaloid formation and storage, research into the detection of this compound in mammals is being questioned. Moreover, the analytical technique for determining a compound in human plasma, urine, cerebrospinal fluid, and the brain requires expensive and sophisticated special devices and therefore is not ideal for routine analyses and is unlikely to become scientifically valuable [16]. To determine whether alcohol has an effect on salsolinol biosynthesis and if it can be a clinical marker to compare alcoholics and non-alcoholics, further experimental work is needed.

#### 3.8.11. Dolichol

Dolichol refers to any members of the group of long chain, which are mostly unsaturated organic compounds that consist of a varying number of isoprene units terminated with an α-saturated isoprenoid group containing an alcohol functional group. It is synthesized from acetate. It accumulates in tissues during aging. Dolichol as a glycosyl carrier is involved in the translational modification of proteins to N-linked glycoproteins. Free radicals, for example, those caused by the consumption of alcohol, influence its function [12,13].

Both dolichol and ethanol are substrates for alcohol dehydrogenase. This means that they compete with each other, and a high level of dolichol in the urine is considered a marker of alcohol abuse [16]. Importantly, thanks to research on humans, we know that moderate alcohol consumption (60 g/day), as opposed to drinking it, does not increase the level of dolichol in the urine. In the infants of alcoholic mothers and in persistent alcoholics, an increased level of urine dolichol was noted. Correlated with urinary creatinine, it was 2.5–4 times higher than in non-alcoholic social alcoholics. Its half-life in urine is about 3 days, and in serum, it is over 7 days, [65,76,77]. High levels of dolichol in mocx returned to normal in alcoholics by day 5 of alcohol abstinence [76]. Although urine testing with dolichol was highly unique (96%), its sensitivity is moderate (68%) and even low (9–19%) [65].

#### 3.8.12. Circulating Cytokines

Cytokines are the messenger molecules of the immune system. They mediate cellular interactions among lymphocytes, dendritic cells, endothelial cells, and CT cells. They are proteins produced by many cell types that modulate the functions of other cell types, which mediate and regulate immune and inflammatory reactions. The majority of cytokines act on the cells that produce them (autocrine), neighboring cells (paracrine), or rarely on distant sites (endocrine). They are involved in inflammation, proliferation, the migration of cells, and regeneration, which are key factors for the development and functioning of adaptive and innate immune system [78].

Acute alcohol indrinking and long-lasting consumption have been shown to affect adaptive immune responses and inflammatory cell responses by reducing the variety of immune reactions. Additionally, alcohol alters cytokine levels in tissues, e.g., brain, lung, liver, and plasma [78,79,80,81]. We can use circulating cytokines as a diagnostic technique for alcohol abuse to accessibly measure the serum levels of numerous cytokines in clinical practice.

The most commendatory candidates are interleukin (IL) -1 a, IL-1b, IL-6, tumor necrosis factor-a (TNF-a) Monocyte, Il-8, and Il-12, chemoattractant protein-1 (MCP-1) [78]. In alcoholics, serum levels of TNF-α are greater compared to those of the general population, regardless of alcohol intake level [82]. Circulating TNF-α, Il-6, and Il-1 were found to be elevated in both persistent and severe alcohol-induced liver illness. Additionally, persistent alcohol consumption without associated liver illness has been related to the notably increased creation of TNF-a, IL-1b, Il-12, and Il-6 [78]. On the other hand, actively drinking people with liver cirrhosis caused by alcohol show unusually low levels of inflammatory cytokines. Intriguingly, no considerable modifications in levels of cytokine were observed in persons with alcohol liver cirrhosis or remained during alcohol abstinence [83]. Despite the tremendous amount of evidence that states the usefulness of circulating cytokines as a sign of alcohol intake, it is not likely that they will be utilized as standard alcohol biomarkers. Likewise, the function of other factors in cytokine release, such as gender, age, nutrition, method of analysis, and comorbid substance abuse, still need to be elucidated in alcohol abusers.

### 3.9. Proteomic Strategies

Proteomics is, in other words, the analysis of many proteins in one sample. The most important opportunity it gives us is the detailed characterization of proteins in a cell, tissue, or organ, which gives us insight into the condition of a given sample. Based on a methodological definition, proteomics deals with the physical arrangement of amino acids in a protein (structural proteomics), the physiological activity of proteins (practical proteomics), and the patterns of protein modification and expression in health and disease (expression proteomics). One of the goals of proteomics is to identify disease biomarkers [84]. The literature gives us clear indications that proteomics methods may be the future of the characterization and validation of new alcohol abuse biomarker panels [85]. Moreover, proteomics experiments can identify an infinite number of proteins in one series. In the non-human primates group, a speculative panel of 17 plasma proteins (including plasma cytokines, developmental elements, and other proteins) adequately classified alcohol abusers with a 100% level of sensitivity and distinguished each level of drinking from alcohol abstinence with 88% accuracy [86].

Human serum proteins, candidates for new alcohol biomarkers, consist of AT-rich phosphatidylcholine-sterol acyltransferase containing the interactive domain, protein 4B, hepatocyte growth factor-like protein, ADP-ribosylation aspect [87], clusters, serum amyloid A4, fibronectin [47], a2-HS glycoprotein, apolipoprotein AI, glutathione peroxidase 3, epithelial pigment origin factor, heparin cofactor II [88], fibrinogen fragment, isoform 1 [89], gelsolin, P-selenoprotein, serotransferrin, hemopexin, and tetranectin [90]. Some studies have also found that an increase in oral peroxidase (OPO) activity in alcohol-dependent individuals due to its reduction in drinkers and reduced immunoglobulin A (IgA) secretion in addicted individuals and increased IgA levels in drinkers suggested the potential use of salivary IgA and OPO in the differential diagnosis of acute and chronic alcohol consumption [91]. Among the salivary glycoproteins of alcohol-dependent people, significant changes were also found in the glycosylation profile of α-amylase, clusterin, haptoglobin, light and heavy chains of immunoglobulins, and transferrin, thus suggesting that these glycoproteins may be potential markers of alcohol dependence in the future [92]

### 3.10. ACE-2 Enzyme

During the severe acute respiratory syndrome coronavirus 2 (SARS-CoV-2) pandemic, alcohol consumption increased markedly. Nearly one in four adults reported drinking more alcohol to cope with stress. Chronic alcohol abuse is now recognized as a factor that complicates the course of acute respiratory distress syndrome and increases mortality. To investigate the mechanisms behind this interaction, a combined acute respiratory distress syndrome and chronic alcohol abuse mouse model was developed by intratracheally instilling the subunit 1 (S1) of SARS-CoV-2 spike protein (S1SP) in K18–human angiotensin-converting enzyme 2 (ACE2) transgenic mice that expressed the human ACE2 receptor for SARS-CoV-2 and were kept on an ethanol diet. Seventy-two hours after S1SP instillation, mice on an ethanol diet showed a strong decrease in body weight, a dramatic increase in the white blood cell content of bronchoalveolar lavage fluid, and an augmented cytokine storm compared with S1SP-treated mice on a control diet. The histologic examination of lung tissue showed abnormal recruitment of immune cells in the alveolar space, abnormal parenchymal architecture, and a worsening Ashcroft score in S1SP- and alcohol-treated animals. Along with the activation of proinflammatory biomarkers (NF-κB, STAT3, NLR family pyrin domain-containing protein 3 (NLRP3) inflammasome), lung tissue homogenates from mice on an alcohol diet showed an overexpression of ACE2 compared with mice on a control diet. This model could be useful for the development of therapeutic approaches against alcohol-exacerbated coronavirus disease (2019) [93] (Table 4).

## 4. Limitations

The study is not systematic and does not provide quantitative information. The authors did not use strict inclusion and exclusion criteria. The authors described markers with specified accuracy to alcohol use detection as well as potential alcohol markers. Both large and small studies have been included in the work to present both well-known alcohol markers and potential markers with yet-unproven efficacy, which should be included in future marker work.

## 5. Conclusions

No trustworthy medical diagnosis of alcohol abuse/dependence can be made from any recognized laboratory marker, even in combination with another marker, unless the patient is medically evaluated. On the other hand, a medical diagnosis can be made without lab tests [94]. For this reason, behavioral evaluation, physical examination, and an interview are the basis for the medical diagnosis of alcohol use. Survey techniques (e.g., CAGE “Cut down, Annoyed, Guilty, morning Eye-opener”, MAST “The Michigan Alcoholism Screening Test”, or AUDIT “Alcohol Use Disorders Identification Test”) and lab tests are useful for verification rather than the basis of the medical diagnosis [95]. The sensitivity and uniqueness of markers depend on the study population, age of participants, sex of participants, diseases connected with alcoholic use, and the physiological state (e.g., pregnancy) of participants. Biological markers need not to be used as the main screening tool for alcohol use.

Because of the many restrictions and weaknesses of the presently used biomarkers of alcohol intake, none of them have become commonly accepted, and the search for suitable (i.e., more sensitive and specific) biomarkers continues. While it may be appealing to think about biomarkers as single particles, a growing body of proof suggests that panels of mixed biomolecules might work best in regard to the level of sensitivity and specificity. Further investigations need to illuminate the crucial pathophysiological bases of alcohol drinking behavior and ethanol-induced organ damage and eventually lead to better forms of prevention and treatment. Notably, future alcohol biomarkers should be able to differentiate between a range of drinking behaviors occurring in everyday practice (abstinence vs. light drinking vs. heavy drinking) instead of just separating between nondrinking and drinking. In addition, they should likewise make the evaluation of both typical and atypical drinking patterns (e.g., chronic and binge drinking) possible.

In populations not previously identified in terms of alcohol issues, it is possible, nevertheless, to use newer markers (e.g., % CDT, 5-HTOL, proteomic markers) due to their high uniqueness, and they can be integrated with a more conventional marker such as GGT for confirmation. In primary healthcare and health center facilities, the screening method may be a combination of% CDT + GGT + AUDIT questionnaire [10]. The most beneficial (sensitive) of the readily available traditional biomarkers utilized in the diagnosis of alcohol use remains GGT, especially when combined with ALT, AST (and De Ritis Index), and MCV [26]. Considering that the determination of ethanol in blood and exhaled air is reputable for a short time, and the boost in GGT, MCV and CDT was noted only after chronic intake of high dosages of alcohol, AST seems to be a beneficial marker in the diagnosis of severe (likewise one-time) intoxication with a high dosage of ethanol. In abstinence monitoring, EtG is an even more sensitive indicator of acute ethanol poisoning than ethanol itself.

The most important challenge in the diagnosis of alcohol use and alcohol use disorders in the future will be the translation of expensive and often advanced analytical techniques for figuring out the selected compounds in quickly available tissue and fluids into affordable and simple diagnostic tools to be utilized in routine medical practice. Larger population studies are also needed to test the usefulness of these new potential markers of alcohol use.

## Figures and Tables

**Table 1:** Summary characteristics of traditional alcohol biomarkers.

Parameter	g-Glutamyl Transferase (GGT)	Alanine Amino-Transferase (ALT)	Aspartate Amino-Transferase (AST)	Carbohydrate-Deficient Transferrin (CDT)	Mean Corpuscular Volume (MCV)
Type of drinking characterized	Probably at least 5 drinks/day for several weeks	Unknown, but heavy and lasting for several weeks	Unknown, but heavy and lasting for several weeks	Probably at least 5 drinks/day for c.a. 2 weeks	Unknown, but heavy and lasting at least a few months
Dose-response of alcohol	80–200 g/day	≥40 g/day	≥40 g/day	>50 g/day	≥60 g/day
Time to elevation	24 h–2 weeks	3–7 days	3–7 days	1–2 weeks	>4–6 weeks
Time to descent to normal levels	2–6 weeks of abstinence (T½ = 14–26 days)	2–4 weeks of abstinence (T½ = 37–57 h)	2–4 weeks of abstinence (T½ = 12–24 h)	2–3 weeks of abstinence (T½ = 15 days)	4 months of abstinence
Sensitivity for detecting excessive alcohol consumption	37–95%	5–40%	25–60%	55–90%	40–50%
Specificity	18–93%	50–57%	47–68%	92–97%	80–90%
Relapse sensitivity	50%	Not reported	Not reported	55–76%	20%
Current clinical use	Identifying chronic alcohol abuse. Screening for heavy drinking. Useful for monitoring abstinence in treatment programs	Identifying chronic alcohol abuse. Screening for heavy drinking	Identifying chronic alcohol abuse. Screening for heavy drinking	Screening for alcohol dependence. Screening for heavy drinking. Identifying relapse (especially to heavy drinking)	Screening for heavy drinking
Strengths in clinical use	High specificity in patients with suspected alcohol abuse. Elevation precedes alcohol induced liver damage. Effective marker for patients suspected of binge drinking. Inexpensive	Highly sensitive and specific for alcohol-induced liver damage	Highly sensitive and specific for alcohol-induced liver damage	High specificity for alcohol use. High sensitivity in distinguishing alcoholics from social drinkers. Confirmatory test for patients suspected of alcohol abuse. Marker of relapse and abstinence from drinking	Accuracy similar in male and female subjects. Indicates chronicity of drinking. Routine laboratory test
Limitations in clinical use	Many factors cause false positives. Poor screening tool in general population (due to low sensitivity). Poor marker of relapse	LT seems to be less sensitive than AST. Enzyme elevation can be detected only after periods of heavy drinking. Elevation secondary to liver damage at hepatocellular level	Enzyme elevation can be detected only after periods of heavy drinking. Elevation secondary to liver damage at hepatocellular level	Low sensitivity; more valuable to confirm than to exclude heavy drinking. Poor screening tool for alcohol use in general population. Cost and low availability of testing	Many factors cause false positives. Poor screening tool for alcohol abuse (due to low sensitivity). Poor marker of relapse

**Table 2:** Factors that influence sensitivity and specificity of traditional biomarkers.

Factor	Influence	
age	Young people	Elderly
	Questionnaire methods better at detecting alcohol abuse. Lower sensitivity of GGT and CDT (8% and 17%) at the age of 21–35 years than aged 36–50 (43% and 57%). Overall, the sensitivity of alcohol biomarkers is low. The reason may be faster return of indicators to normal (relatively low level of consumption, faster elimination).	Less usefulness of questionnaires. At age >51 years, lower sensitivity for CDT (46%), higher for GGT (58%). The overall sensitivity of the alcohol biomarkers is greater (slower elimination, higher levels of consumption?).
sex	men	women
	The level of increase in GGT depends on the intensity of drinking. The level of increase in CDT depends on the frequency of drinking.	The level of increase in GGT and CDT depends more on the intensity (amount of alcohol g/day) than on the frequency (number of days of consumption) of drinking.
	Factors such as: - Differences in the intensity and frequency of drinking (higher in men), - Physiological factors (higher alcohol level in women than in men after drinking the same dose of alcohol may result from: lower water content in the body of women–lower distribution, lower activity of gastric alcohol dehydrogenase in women), - Sociological.	
liver diseases	- Alcohol abuse is present in 20–90% of liver diseases and 50% of cirrhosis. - Single traditional tests rarely differentiate alcoholic liver disease from non-alcoholic liver disease (i.e., GGT and CDT increase in both). - CDT is more specific than GGT in alcohol abuse. - The De Rittis Index (AST/ALT) is the most effective of the available biomarkers in differentiating alcohol from non-alcoholic liver injury; a value >1.5 is suggestive, and a value >2 is almost a confirmation of the alcoholic etiology of a liver injury.	
pregnancy	- Between 14% and 20% of pregnant women consume alcohol at some point during their pregnancy. - Fetal Alcohol Spectrum Disorder (FASD) affects approximately 1% of newborns. - No single biomarker used, even in combination with another marker, has been put into practice in the clinical detection of alcohol abuse in pregnant women. - The increase in two or more markers (especially GGT, MCV, and CDT) seems to be a more sensitive method of FASD prevention than interview and questionnaire studies. - A combination of markers with the questionnaire method in the detection of abuse is also recommended.	

**Table 3:** Biomarkers of alcohol abuse with short, intermediate, and long half-lives. Short-range, mid-range, and long-range biomarkers of alcohol abuse. EtOH, ethanol; AcAld, acetaldehyde; 5-HTOL, 5-hydroxytryptofol; 5-HTOL/5-HIAA, 5-hydroxytryptofol/5-hydroxyindole acetic acid indicator; FAEEs, fatty acid ethyl esters–fatty acid ethyl esters; EtG, ethyl glucuronide–ethyl glucuronate; PEth, phosphatidyl ethanol–phosphatidylethanol; EtS, ethyl sulfate–sulfuric acid ethyl esters; AA, acetaldehyde adducts–acetaldehyde adducts; WBAA–whole-blood-associated acetaldehyde; HAA–hemoglobin-associated acetaldehyde; β-HEX, β-hexosaminidase; SA, sialic acid–sialic acid; MCV, mean corpuscular volume–mean red blood cell volume; GGT, gamma-glutamyl transferase; AST and ALT, -aspartate and -alanine aminotransferases; CDT, carbohydrate-deficient transferrin–low-carbohydrate isoforms of transferrin.

Biomarker	Time of Detection	T½	Clinical Characteristic		Reference
EtOH	short	1 h/1 h/10 kg from blood	Measured in blood, expired air, urine, and saliva. Routine determination of serum alcohol concentration >1‰, >1.5‰ without obvious signs of intoxication, and > 3‰ in all circumstances, point to alcohol dependence. Used in emergency departments. For heavy drinkers, elimination is about 1.5 times faster than for social drinkers and risky drinkers. Detectable in urine several hours longer than in blood.	Mainly indicators of alcohol poisoning	[2,16,19,22,23,34]
AcAld	Short	Not far longer than ethanol	Little and short-term diagnostic usefulness.	Mainly indicators of alcohol poisoning	[16,22]
Acetic acid	Short	Not far longer than ethanol	Little and short-term diagnostic usefulness.	Mainly indicator of alcohol poisoning	[16,22]
Methanol	Short	Few hours	Increases after prolonged use of alcohol	Indicator of current intoxication and recent consumption	[16,36]
5-HTOL	Short	14–15 h	Determined in urine. A responsive rate of drinking relapse. The 5-HTOL/5-HIAA ratio increases the specificity.	Indicator of current intoxication and recent consumption	[20,21,37]
FAEEs	Medium	22–44 h	Detectable in serum after occasional drinking. Detectable in hair for several months–marker of chronic consumption.	Mainly indicator of recent consumption	[18,20,38]
EtG	Medium	3–5 days	Determination in urine. In the blood, up to 36 h. In hair, longer. In the USA, it is used commercially in addiction treatment programs. A more sensitive indicator of acute ethanol poisoning than ethanol itself.	Mainly indicators of heavy drinking	[20,38,39]
PEth	Medium	2 weeks	Specific metabolite of ethanol. Increases mainly after prolonged consumption. Sensitive to probe storage.	Mainly indicator of heavy drinking	[7,23]
EtS	Medium	16–27 h longer than ethanol	Determined in urine. It is only detectable in people who drink alcohol (even in small amounts)	Mainly indicator of heavy drinking.	[38,39]
AA	Medium	Depends on bound protein (with WBAA 3 weeks)	Acetaldehyde bound to blood proteins (WBAA) incl. with hemoglobin1 (HAA) and tissue proteins. It forms the so-called adducts, which, as neoantigens, stimulate the growth of immunoglobulin A in alcohol-dependent persons.	Mainly indicator of heavy drinking	[2,16,40,41]
β-HEX	Long	7–10 days (plasma) 4 weeks (urine), ~4 weeks of β-HEX A isoenzyme in saliva	In the detection of chronic alcohol consumption. Lowering specificity of hypertension, diabetes, liver disease, hyperthyroidism, pregnancy, oral contraceptive drugs, stroke, and myocardial infarction.	Mainly indicator of heavy drinking	[40,42,43]
SA	Long	2–5 weeks	Tender to alcohol abuse but low specificity. Helpful in distinguishing alcohol abuse from secondary disorders in liver disease. In prolonged consumption, a decrease in the sialic acid index of the SIJ apolipoprotein.	Mainly indicator of heavy drinking	[44,45]
MCV	Long	2–4 weeks	More applicable to women. Specificity is reduced by vitamin B6, B12, or folate deficiency, liver disease, hypothyroidism, hematological diseases, reticulocytosis, and smoking.	Mainly indicator of heavy drinking	[2,4,23,46]
GGT	Long	2–3 weeks	A sensitive and cheap test. Does not increase after acute alcohol poisoning. The specificity is reduced by obesity, diabetes, non-alcoholic liver diseases, pancreatitis, hyperlipidemia, heart failure, massive injuries, drugs (barbiturates, antiepileptic drugs, anticoagulants), kidney diseases.	Mainly indicator of heavy drinking	[2,4,23,28,46]
AST, ALT	Long	2–3 weeks	A De Ritis Index (AST/ALT). >1.5 suggests, and >2 is almost a confirmation of alcohol-related liver injury. Possible increase in AST after acute poisoning with a large dose of alcohol. The determination of the mitochondrial isoenzyme mAST increases the specificity.	Mainly indicator of heavy drinking	[2,4,25,30,31,46]
CDT	Long	2–3 weeks	The most specific marker available today. The specificity is reduced by rare genetic defects in transferrin. It is the only test that has been approved in the USA by the FDA (Food and Drug Administration) for the identification of heavy drinking.	Mainly indicator of heavy drinking	[29,32,33,38,45,47]

**Table 4:** New biomarkers of alcohol abuse.

Biomarkers	Data
Ethyl glucuronide (EtG) and ethyl sulfate (EtS)	EtS and EtG are premium indicators of current alcohol intake of a longer recognition span. Particularly, EtG exists in blood for approximately 36 h (in comparison with complete elimination of ethanol from the blood −8 h) and in urine for 3–5 days after hefty alcohol intake, whereas EtS is noticeable in urine c.a. 16–27 h longer than ethanol [39].
Acetaldehyde, acetaldehyde adducts, and anti-adduct antibodies	Complying with alcohol consumption, concentration of free acetaldehyde is very changeable with a life expectancy of c.a. 3 h; however, some acetaldehyde-protein adducts might be found approx. 3 weeks after alcohol intake, and hemoglobin-bound acetaldehyde (HAA) gathers in red blood cells during their 120-day typical lifespan. A singular high amount of alcohol (2 g/kg) boosts blood HAA when traditional markers, such as GGT or MCV, reveal no difference [41,51].
Fatty acid ethyl esters	Fat ethyl esters are used as postmortem indicators of alcohol consumption. FAEEs found accumulated in the hair are a very promising marker of a person’s drinking habits because they cannot be rinsed out of the hair and accumulate with alcohol consumption.
Phosphatidylethanol	In vitro studies showed that the amount of PEth in human red blood cells was directly proportional to ethanol concentration and exposure time, and there was no correlation between the rate of PEth formation and hematological indices (i.e., MCV, red blood cell count, hematocrit). Moreover, there is no enzymatic degradation of PEth in human erythrocytes, so it is deposited in cell membranes, suggesting a potential utility in measuring long-term or binge alcohol consumption
β-hexosaminidase	It was established that the diagnostic sensitivity levels of increased β-HEX B activity in serum and β-HEX in urine are 69–94% and 81–85%, respectively. In addition, in alcohol-dependent people, the level of enzymes drops dramatically during the sobriety period (7–10 days, T½ = 6.5 days) [64,65]. However, levels of β-HEX in saliva, urine, or serum may also increase after isolated ingestion of about 2 g/kg of alcohol (also known as ‘binge drinking’) [66].
Plasma Sialic Acid Index of Apolipoprotein J	As with molecular transferrin, long-term ethanol poisoning reduces plasma Apo J sialylation, primarily by increasing sialidase activity and reducing cellular glycosyltransferases (i.e., 91). SIJ is reduced in alcoholics (typically by 50–57%, with a specificity of ~100%), and levels gradually return to the normal range over the course of several weeks of abstinence from alcohol (T½ = 4–5 weeks) [63,68]. Moreover, plasma SIJ is associated with relapses in people addicted to alcohol (sensitivity ~ 90%) [13].
Total serum sialic acid	Compared to social drinkers, alcoholics have increased amounts of TSA in saliva, urine, and serum, although the exact mechanisms by which it occurs are debated [13,69,70]. The diagnostic value of TSA as a biomarker of alcohol abuse showed a level of sensitivity of 48–58% and a specificity of 64–96% [13,69].
Cholesteryl ester transfer protein	According to the literature, CETP activity and its plasma concentration are minimized by alcohol consumption. This leads to a diversion of the cholesteryl esters and an increase in plasma HDL levels (a frequent laboratory peculiarity in people addicted to alcohol) [13,28]. The potential scientific value of CETP in plasma is believed to be similar to traditional alcohol markers such as AST, ALT, GGTP, MCV.
5-Hydroxytryptophol, 5-hydroxyindole-3-acetic acid	Alcohol and the main metabolite of its oxidation, acetaldehyde, affect the metabolism of serotonin in such a way that the level of 5-HTOL rises after drinking alcohol. Elevated levels of 5-HTOL persist in urine for 5–15 h (depending on dose) after alcohol consumption compared to baseline measurements that suggest that ethanol persists longer in urine than in blood [37].
Salsolinol	It has been shown that the total amount of salsolinol in urine and the concentration of salsolinol in plasma change differently after acute alcohol consumption. Alcohol consumption showed a significant increase in both urine and plasma levels. Moreover, compared with non-alcoholics, alcoholics who abstained for only 1 week had decreased levels of salsolinol in one type of white blood cell (especially lymphocytes) [74].
Dolichol	Both dolichol and ethanol are substrates for alcohol dehydrogenase. This means that they compete with each other, and a high level of dolichol in the urine is considered a marker of alcohol abuse [16].
Circulating cytokines	Acute alcohol drinking and long-lasting consumption show to affect adaptive immune responses and inflammatory cell responses by reducing variety of immune reactions. Additionally, alcohol alters cytokine levels in tissues, e.g., brain, lung, liver, and plasma [78,79,80,81]. We can use circulating cytokines as a diagnostic technique for alcohol abuse to accessibly measure the of serum levels of numerous cytokines in clinical practice.
proteomic strategies	Human serum proteins, candidates for new alcohol biomarkers, consist of AT-rich phosphatidylcholine-sterol acyltransferase containing the interactive domain, protein 4B, hepatocyte growth factor-like protein, ADP-ribosylation aspect [87], clusters, serum amyloid A4, fibronectin [47], a2-HS glycoprotein, apolipoprotein AI, glutathione peroxidase 3, epithelial pigment origin factor, heparin cofactor II [88], fibrinogen fragment, isoform 1 [89], gelsolin, P-selenoprotein, serotransferrin, hemopexin, and tetranectin [90].
ACE-2 enzyme	Along with the activation of proinflammatory biomarkers (NF-κB, STAT3, NLR family pyrin domain-containing protein 3 (NLRP3) inflammasome), lung tissue homogenates from mice on an alcohol diet showed an overexpression of ACE2 compared with mice on a control diet.

## Data Availability

There were no new data created.
